# Association of Narrow Anterior Communicating Artery or Contralateral A1 Segment with Poor Outcomes After Mechanical Thrombectomy

**DOI:** 10.3390/medicina60111749

**Published:** 2024-10-24

**Authors:** Audrius Širvinskas, Giedrius Ledas, Rūta Levulienė, Jurgita Markevičiūtė, Valerija Mosenko, Andrej Afanasjev, Aleksandras Vilionskis, Saulius Lukoševičius, Algirdas Edvardas Tamošiūnas

**Affiliations:** 1 Department of Radiology, Republican Vilnius University Hospital, Šiltnamių g. 29, 04130 Vilnius, Lithuania; 2 Department of Radiology, Nuclear Medicine and Medical Physics, Institute of Biomedical Sciences, Faculty of Medicine, Vilnius University, 03101 Vilnius, Lithuania; valerijamosenko@gmail.com (V.M.); algirdas.tamosiunas@santa.lt (A.E.T.); 3 Institute of Applied Mathematics, Vilnius University, 03225 Vilnius, Lithuania; ruta.levuliene@mif.vu.lt (R.L.); jurgita.markeviciute@mif.vu.lt (J.M.); 4 Clinic of Neurology and Neurosurgery, Institute of Clinical Medicine, Faculty of Medicine, Vilnius University, 04130 Vilnius, Lithuania; aleksandras.vilionskis@rvul.lt; 5 Medical Academy, Lithuanian University of Health Sciences, 44307 Kaunas, Lithuania; saulius.lukosevicius@lsmu.lt

**Keywords:** hypoplasia, contralateral A1, anterior communicating artery, stroke outcome, mechanical thrombectomy, NIHSS, ASPECTS

## Abstract

*Background and Objectives:* Contralateral A1 and AComA aplasia/hypoplasia are critically important in distal ICA T occlusion as the protective collateral blood supply from the circle of Willis via the anterior communicating artery is compromised. Although the terms aplasia/hypoplasia are used broadly in the literature, the need for concrete measurements and data on their clinical significance is apparent. Features of the individual anatomy of the circle of Willis may determine patient outcomes. We aim to determine the cut-off values of contralateral A1 and AComA segments that determine worse outcomes for patients with acute ischemic stroke with T occlusion of the terminal internal carotid artery. *Material and Methods:* Retrospective patient data from 2015 to 2020 and prospective data from 2021 to 2022 of 482 patients with diagnosed acute ischemic stroke that underwent mechanical thrombectomy at the Republican Vilnius University Hospital (Vilnius, Lithuania) were obtained. Of these patients, 70 were selected with occlusion of internal carotid artery bifurcation and extension to M1 or A1 segments. For statistically significant interactions, patient data were analyzed using two statistical methods (logistic regression and Multivariate Adaptive Regression Splines (MARS)). *Results:* The narrowest segment of contralateral A1 and/or AComA was statistically significant for 7-day NIHSS, and the optimal cut-off points for this variable were 1.1 mm (MARS model) and 1.2 mm (logistic regression, *p* = 0.0079, sensitivity 66.7%, specificity 67.9%). The other considered variables (age, gender, time from last seen well to groin puncture, intravenous recombinant tissue plasminogen activator, admission NIHSS, and ASPECT score) and their interactions were not statistically significant. *Conclusions:* A negative correlation was found between the narrowest segment and seven days of NIHSS. A larger diameter of contralateral A1 and AComA appears to be essential for better patient outcomes at 7-day evaluation post mechanical thrombectomy.

## 1. Introduction

In the era of mechanical thrombectomy (MTE), leptomeningeal collaterals have been shown to play a pivotal role in brain tissue survival and patient outcomes [1,2]. In a significant proportion of patients with anterior circulation stroke, the distal part of the carotid artery is occluded, and the circle of Willis (CoW) becomes an essential pathway for collateral cerebral blood flow [3]. Studies have shown that an insufficient anterior communicating artery (AComA) or contralateral anterior cerebral artery (ACA) A1 segment (cA1) diameter may impair collateral brain circulation in different types of distal internal carotid artery (ICA) occlusions—the I type, involving the C7 segment of the carotid artery, the L type, involving the C7 and M1 segments, and the T type, involving the C7, M1, and A1 segments [4,5,6].

The published literature presents different criteria for CoW collateral insufficiency. One commonly used cut-off for narrow AComA and ACA A1 is 1 mm; however, some authors use various “functional” definitions [4,5,6]. Fischer et al. found that functional aplasia of the cA1 in distal ICA occlusion was associated with poorer results in patients undergoing MTE [4].

To date, the literature lacks data regarding the effect of CoW configuration on patient outcomes after mechanical thrombectomy. This study aimed to approximate the diameter of the anterior part of the circle of Willis that could affect patient outcomes with distal ICA T occlusion after MTE.

In distal ICA T occlusion, when a blood clot is blocking the C7, M1, and A1 segments, blood is brought through the contralateral A1 and anterior communicating artery to the ipsilateral ACA territory—essential as a source of collateral circulation for the MCA territory. In this study, we describe the cA1 and AComA complex as a continuum essential for collateral blood supply, as only the narrowest part of this complex is important hemodynamically. The AComA/A1 complex could be vital for collateral circulation to the ipsilateral MCA via the ipsilateral ACA.

## 2. Materials and Methods

### 2.1. Patients

Data from 482 patients with acute ischemic stroke that underwent MTE at the Republican Vilnius University Hospital (Vilnius, Lithuania) were analyzed. Patient-informed consent for prospective patient data was obtained in written form, and for retrospective cases from the local patient database, the hospital bioethics committee waived this requirement. Permission from the local bioethics committee was obtained (No. 2021/2-1315-791 24 August 2023).

Retrospective patient data from 2015 to 2020 and prospective data from 2021 to 2022 were gathered. Retrospective patient data comprised 320 patients, and prospective patient data comprised 162. Of these patients, 35 were selected from the retrospective and 35 from the prospective patient groups. 

The inclusion criteria were as follows: (1) age >18 years old, (2) occlusion of internal carotid artery bifurcation with extension to either the M1 or A1 segments on preprocedural computed tomography angiography, (3) successful mechanical thrombectomy defined by final recanalization of a modified Thrombolysis in Cerebral Infarction (mTICI) score of 2b or 3, and (4) patient consent. 

The exclusion criteria were as follows: (1) mechanical thrombectomy defined by final recanalization of a modified Thrombolysis in Cerebral Infarction (mTICI) score of 2a or lower, (2) distal occlusions in the A2 and A3 or M3 and M4 segments, (3) patients with previously performed stenting of the intracranial or extracranial vessels and/or the preprocedural usage of antiplatelet drugs, and (4) an A1 segment thrombus extending to or narrowing the anterior communicating artery–A1 junction on preprocedural computed tomography angiography.

The following data from patients were gathered: gender, age, time from last seen well to punction, intravenous recombinant tissue plasminogen activator, narrowest segment of artery, primary arterial hypertension, heart failure, diabetes mellitus, hemorrhage on follow-up CT, ASPECT score, admission NIHSS, modified Rankin Scale score after 90 days, and hemorrhagic transformation of ischemic stroke. Data on the primary arterial hypertension, heart failure, diabetes mellitus, and hemorrhage on follow-up CT variables were available only for the some of the patients. 

**Figure 1:**
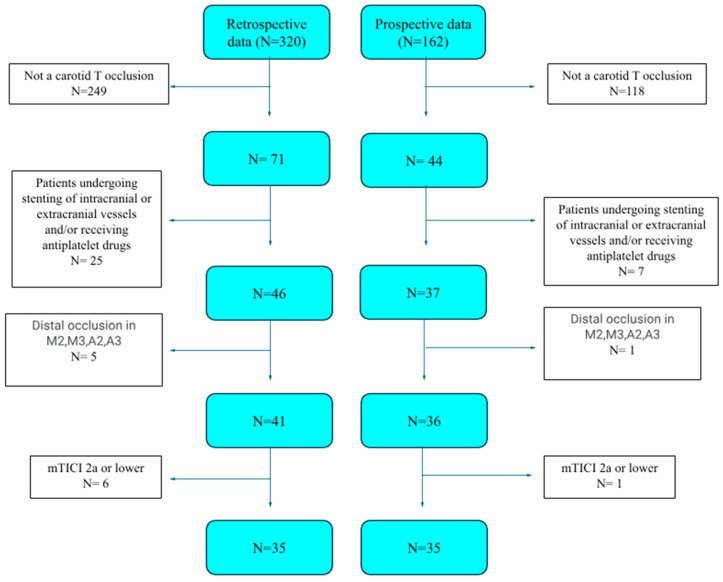
Patient selection flowchart.

The patient selection flowchart is presented in Figure 1. 

### 2.2. Imaging Protocol

Patients with suspected stroke were evaluated by emergency room neurologists using a standard protocol of the National Institutes of Health Stroke Scale (NIHSS) score and were assigned to urgent head CT/CTA.

All CT/CTA images were acquired using a 64-slice computed tomography (CT) machine, Aquilion TSX-101AQC; serial nr: QCA 11Z2080 2426; TOSHIBA, Tokyo, Japan, with the following parameters: a 120 kVp tube voltage, a 200 mA tube current, and a 0.5 mm slice thickness. CTA images were reconstructed with a 0.5 mm slice thickness. The contrast agent used for CTA was Iohexol 350 mg I/mL (Omnipaque; GE Healthcare, Oslo, NO, USA) with a standard 80 mL dose (corrected if the patient size was exceedingly small or large) injected intravenously at 4 mL/s. A technologist manually activated scanning when the enhancement at the monitoring site reached 100 HU. The monitored region was both sides’ common carotid artery or internal carotid artery region, depending on the patient’s size and neck position. The arterial phase was scanned from the aortic arch to the vertex, and the venous phase was scanned from the vertex to the skull base.

The extent of acute ischemic changes on non-enhanced CT images was evaluated using the Alberta Stroke Program Early CT Score (ASPECTS). The values of ASPECT scores were assessed for all patients and taken from the hospitals’ Electronic Health Records.

CTA images were assessed by two radiologists (A.Š., fifteen years of experience, and G.L., five years of experience). The diameter of the circle of Willis arteries was assessed using Multiplanar Reconstructions (MPRs) in the projection where the diameter of the vessel was visible best. If the vessel diameter difference between observers was less than 1 mm, the mean was calculated by arithmetic division. In contrast, for more significant differences, the vessel diameter was settled by a live discussion. The interoperator agreement was 0.95 (kappa = 0.95). 

A follow-up CT was performed 24 h after MTE.

### 2.3. Mechanical Thrombectomy

After CT examination, patients suitable for mechanical thrombectomy under the locally approved hospital protocol were referred to the Angiosuite. MTE was performed using one of the commercially available stent retrievers with a distal aspiration catheter or aspiration catheter alone; the device was chosen at the operator’s discretion. Final recanalization was confirmed utilizing the Modified Thrombolysis in Cerebral Infarction (mTICI) score [7]. Successful recanalization was defined as achieving a 2C/3 mTICI score. After the initial attempt, the first-pass effect was characterized by achieving a 2B/3 mTICI recanalization using the selected thrombectomy device.

The procedure was performed under general anesthesia.

### 2.4. Outcomes

A ward neurologist assessed the NIHSS score using the standard NIHSS protocol 12 and 24 h after MTE. Then, 7-day NIHSS was determined based on the clinical data available from patient history. A predicted good outcome was defined as an NIHSS score of 6 or less after seven days, according to the model of Lai et al. [8]. The data were analyzed to find variables leading to a good outcome (seven-day NIHSS after MTE lower or equal to six). The patient mortality rate was calculated based on the value 6 for mRS and the PH2 type of hemorrhagic transformation. Data for individual groups were not available, so the overall mortality rate was calculated. One patient with a subarachnoid hemorrhage due to a ruptured aneurysm was excluded from the hemorrhagic transformation analysis.

### 2.5. Statistical Analysis

Two approaches were selected for the seven-day NIHSS modeling, as follows: logistic regression and Multivariate Adaptive Regression Splines (MARS) [9,10]. The seven-day NIHSS was transformed into a binary variable for the logistic regression model, where values lower and equal to six denote good outcomes and values greater than six denote bad outcomes. In the case of the MARS model, an untransformed seven-day NIHSS was used as the response. 

Statistical significance set at *p* < 0.05. R (version 4.3.2; The R foundation, Vienna, Austria) was used for the analysis. The R function glm from the package stats was applied to fit the logistic regression model, and the MARS model was obtained employing the function train of the package caret.

## 3. Results

### 3.1. Baseline Patient Characteristics

Table 1 represents the summary statistics of the considered variables. The groups of bad and good outcomes were compared using the Fisher exact test in the case of categorical variables, and there was no significant difference at the level of 0.05 between groups for the considered categorical variables (see Table 1). The only value differing between the good and bad outcome groups was the narrowest segment of the AComA/cA1 complex (1.56 ± 0.63 vs. 1.13 ± 0.58, respectively). 

### 3.2. Data Distribution

**Figure 2:**
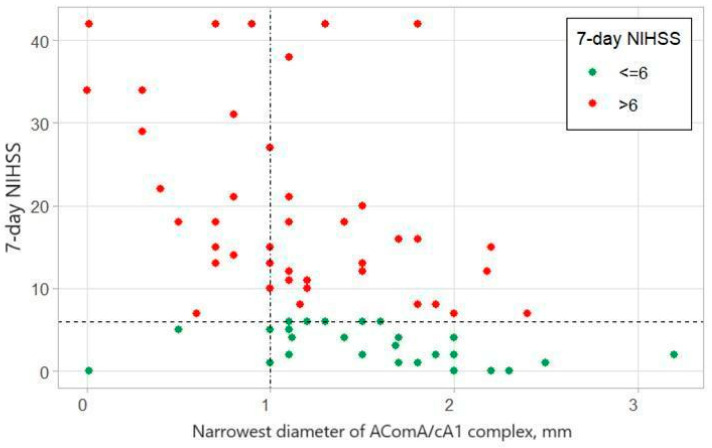
Shows the relationship between the narrowest diameter of the AComA/cA1 complex and the 7-day NIHSS. The graph shows that for values of the narrowest diameter larger than 1 mm, around half of the patients (n = 22) had good outcomes, while when the narrowest diameter was 1 mm or smaller, most patients had poor outcomes.

Logistic regression demonstrated that cA1/AComA being narrower than 1.2 mm leads to worse patient outcomes (NIHSS after seven days <6) after distal ICA occlusion treatment with MTE (*p* < 0.05, OR 0.29) (Figure 2). The MARS model defined the cut-off value for good patient outcomes at 1.1 mm. 

**Figure 3:**
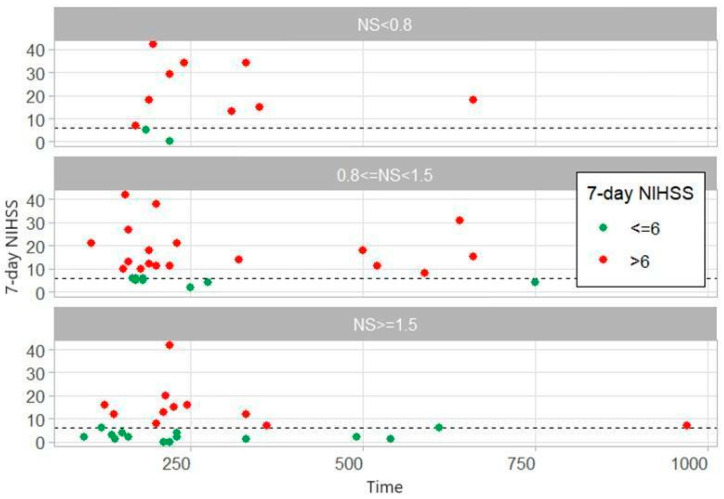
Relationship of time from last seen well to groin puncture and 7-day NIHSS for different narrowest segment (NS) values according to our data distribution in time to groin access vs. seven days NIHSS graph when patients were divided into three groups based on the diameter of the narrowest segment of AComA/cA1 complex (NS < 0.8, 0.8 ≤ NS < 1.5, NS ≥ 1.5). Multivariate analysis did not show any statistically significant difference between narrowest segment groups.

Patients were divided into three groups based on the diameter of the narrowest segment of AComA/cA1 complex:NS < 0.8, 0.8 ≤ NS < 1.5 and NS ≥ 1.5 (Figure 3). In narrowest segment groups with >0.8 mm there were patients with long time from last seen well to groin puncture (>250 min) and good outcome (7-day NIHSS ≤ 6). It seems that good outcomes can be had in patients with a long time to groin access if the narrowest segment of the AComA/cA1 complex is ≥0.8 mm. Further studies of larger study size need to be conducted to determine the impact of the NS for seven days NIHSS with long-to-groin access.

**Figure 4:**
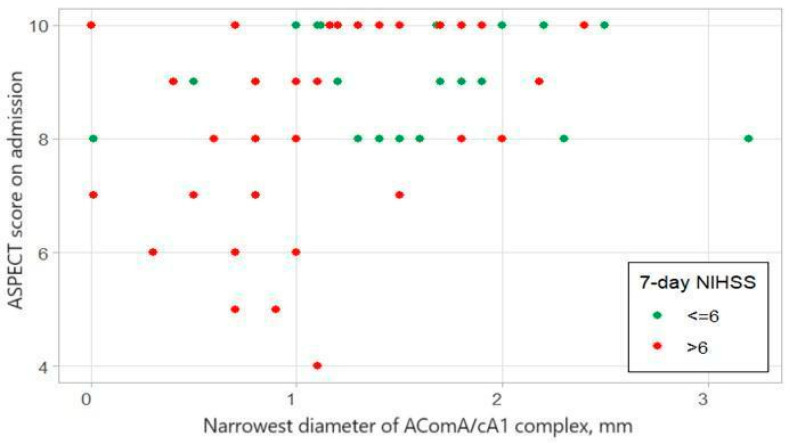
Relationship between narrowest AComA/cA1 complex diameter and ASPECT score on admission.

We analyzed the relationship between narrowest AComA/cA1 complex diameter and ASPECT score on admission for patients with good (7-Day NIHSS ≤ 6) and bad outcomes (7-Day NIHSS > 6) (Figure 4). ASPECT score has a low correlation with the Narrowest diameter of AComA/cA1 complex (Spearman correlation coefficient: 0.3697, *p*-value = 0.0016).

**Figure 5:**
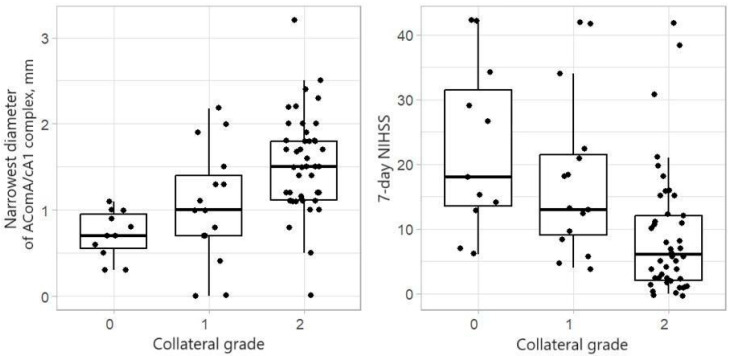
Shows Boxplots of the narrowest diameter of the AComA/cA1 complex, in mm (**left**) and 7-day NIHSS (**right**), by the collateral grade (measured by the Miteff scale).

The boxplots in Figure 5 show that for a more significant collateral grade (measured on the Miteff scale), the median of the narrowest segment is large and the median of the 7-day NIHSS is smaller. 

The Kruskal–Wallis test was used to compare the collateral grade groups, and the Wilcoxon rank-sum test performed pairwise comparisons of the groups. Table 2 presents the obtained *p*-values. For the collateral grade group 0, the narrowest segment of the AComA/cA1 complex diameter median was 0.7 mm and the 7-day NIHSS median was 18. Collateral grade groups 1 and 2 had narrowest diameter medians of 1 mm and 1.5 mm and 7-day NIHSS medians of 13 and 6, respectively. There was no significant difference between the collateral grade groups 0 and 1 for the narrowest AComA/cA1 complex segment. However, the collateral grade groups 0 and 2, as well as 1 and 2, differed significantly. The same result was obtained for the 7-day NIHSS.

Additional data relationships were analyzed: between Time from the last well seen to groin access and the 7-day NIHSS; between Time from the last well seen to groin access and the 7-day NIHSS; between ASPECT score on admission and 7-day NIHSS; between ASPECT on score admission and 7-day NIHSS; between baseline NIHSS and 7-day NIHSS; between the Narrowest diameter of the AComA/cA1 complex and the Baseline NIHSS. No statistically significant data interactions were found. Figures for mentioned relationships can be found in Supplementary Materials.

### 3.3. Data Analysis

#### Modeling the Binary Seven-Day NIHSS

Logistic regression was used to model the probability of bad outcomes (seven-day NIHSS larger than six). Five covariates (age, gender, time (time from last seen well to groin puncture, in minutes), IVrtPA (intravenous recombinant tissue plasminogen activator, Yes/No), and NS (narrowest segment of the anterior communicating artery and contralateral A1 segment, in mm)) were considered. Firstly, univariate logistic regression models were obtained to identify significant factors. Table 2 shows that the only considerable covariate was the narrowest diameter segment of the AComA/cA1 complex. Moreover, the multivariate logistic regression model took five considered covariates and their interactions. Stepwise regression was applied to select the significant effects, and it was obtained that only one variable, the narrowest diameter segment of the AComA/cA1 complex, was significant at the 0.05 level, with an AUC = 0.7066 and cross-validated AUC = 0.6718. The results of logistic regression are given in Table 3. The probability of a bad outcome decreased when the value of the narrowest segment increased. The odds ratio shows that the odds of a worse outcome decreased by 61% when the narrowest segment increased by 1 mm.

Only one variable, the narrowest AComA/cA1 complex segment, was significant. The MARS model suggested the cut-off for this variable was 1.1. Thus, the data and model allow us to conclude that, if the narrowest segment is smaller than the cut-off, which is 1.1, then the outcome for the patient is always bad—the forecast of the seven-day NIHSS is 15. When the narrowest segment is bigger than 1.1, the seven-day NIHSS is predicted to be smaller than 15. Indeed, the larger the value of the narrowest segment, the smaller the value of the seven-day NIHSS. A larger diameter of A1 or AComA leads to better patient outcomes at 7-day evaluation.

## 4. Discussion

Several authors have addressed the importance of AComA/cA1 function in carotid T occlusions for patient outcomes. Fisher et al. described worse outcomes in patients with distal ICA occlusion with functional aplasia of cA1 [4]. Zhao et al. pointed out that patients with a present anterior communicating artery and distal ICA occlusion achieved better outcomes than those without it. Among patients lacking the anterior communicating artery, 94.11% experienced unfavorable outcomes [11]. The importance of the contralateral agenesis of A1 for ICA terminus occlusion is supported by the study of Lee et al. [5]. The morphology of distal ICA occlusions also matters—multivariate analyses showed that functional T occlusion strongly predicted both revascularization success and patient outcomes [6]. Occluded distal ICA with the thrombus extending to A1 in the presence of aplasia or hypoplasia of contralateral A1 or AComA causes ischemic changes in two vascular territories. On the contrary, distal ICA L occlusion, defined as hypoplasia/aplasia of the ipsilateral A1 and distal ICA occlusion, causes ischemic changes in only one vascular territory. All the authors point out that ischemic changes in more than one vascular territory cause poorer patient outcomes [3].

We did not find a study that measured the impact of the narrow AComA/cA1 complex on patient outcomes. Fischer et al. found that functional aplasia of cA1 in distal ICA occlusion was associated with worse results in patients undergoing MTE [4]. We form a hypothesis that the narrowest diameter of the cA1 and AComA complex impacts the number of ischemic stroke territories. As two vascular territories are affected, ischemic stroke should progress faster, have a larger ischemic core, and have a worse prognosis.

In our data, the narrowest segment diameter of the AComA/cA1 complex and collateral grade were statistically important for patient outcomes.

Our data suggest that the discrete diameters of the anterior CoW arteries play an essential role in providing temporary perfusion for the MCA territory until recanalization with MTE is achieved. For values of the cA1 and AComA complex narrowest diameter larger than 1 mm, half of the patients (n = 22) had good outcomes (Figure 2). Logistic regression demonstrated that cA1/AComA being narrower than 1.2 mm leads to worse patient outcomes (NIHSS after seven days < 6) after distal ICA occlusion treatment with MTE (*p* < 0.05, OR 0.29). The MARS model defined the cut-off value for good patient outcomes at 1.1 mm. Two statistical models (logistic regression and Multivariate Adaptive Regression Splines) were used to determine the cut-off more precisely and avoid any statistical error due to the small sample size.

The narrowest diameter of the AComA/cA1 complex is the limit that defines whether one or more vascular territories are affected by ischemic changes. If the AComA diameter is sufficient—larger than 1.1–1.2 mm—distal ICA occlusion is regarded as one vascular territory occlusion, as the ipsilateral ACA territory is sufficiently perfused via collateral circulation.

Our study shows that the median of the narrowest segment of the AComA/cA1 complex was greater in collateral grade value group 2, with statistically significant differences in the diameter between collateral grade groups compared to the collateral grade groups 0 and 1 (Table 2). In our study, patients with collateral grade 2 had statistically significantly better outcomes than grades 0 and 1 (Figure 5, Table 2). Studies have shown that a larger collateral grade value leads to better patient outcomes in occlusive stroke, since ischemic brain tissue is perfused through alternative pathways [12]. Our study has also shown that the narrowest segment of the cA1/AComA complex impacts the collateral grade. Collateral grading is tricky—good contrast phases must be performed, which is sometimes challenging in patients with an altered mental status and unstable hemodynamics. Moreover, collateral grading is subjective. Measurement of the narrowest AComA/cA1 complex segment could be a more objective factor. As it seems, with a sufficiently wide narrowest point of the segment, the collateral grade should also be appropriately high.

In our study, the ASPECT score did not differ statistically significantly between the good and bad outcome groups (*p* = 0.3150) (Table 1). This is contradictory to the MR CLEAN study [13].

**Figure 6:**
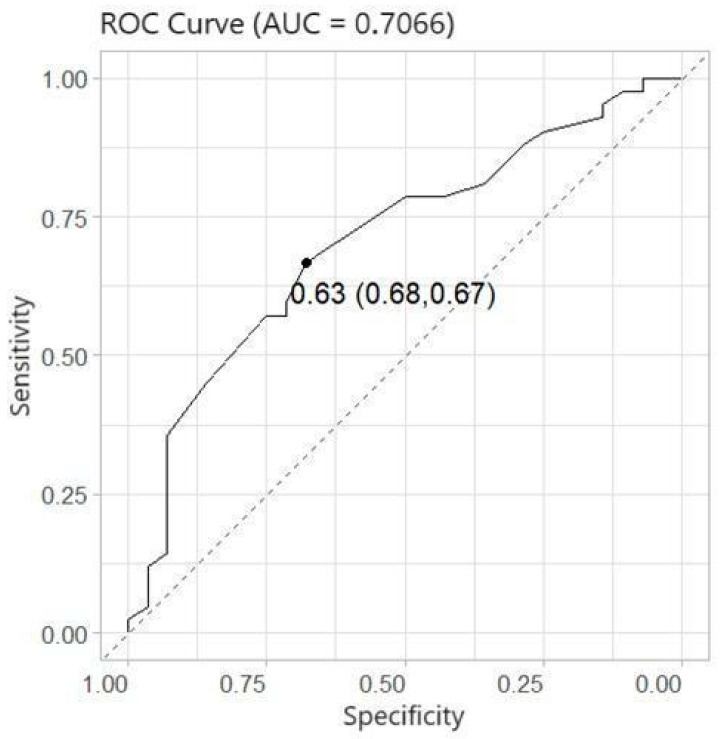
The ROC curve. The optimal probability *p* is based on the Youden index, with corresponding sensitivity and specificity values.

Contrary to this, the narrowest diameter of the AComA/cA1 complex has a statistically significant correlation with patient outcomes, predicting a 66.7% sensitivity and 67.9% specificity (Figure 6). In the case of distal ICA occlusion, the narrowest diameter of the AComA/cA1 complex can be a more important prognostic factor than the ASPECT score, especially in early stroke diagnosis, when the ischemic core is not yet formed on native CT and the ASPECT score is high. Interestingly, the ASPECT score on admission has a low correlation with the narrowest diameter of the AComA/cA1 complex (Spearman correlation coefficient: 0.3697, *p*-value = 0.0016) (Figure 4). We theorize that the ASPECT score is insufficient for determining a poor patient prognosis. ASPECT scoring is also subjective and can differ between two evaluators.

Our study limitations include a partial retrospective design, a small sample size, and the lack of functional assessment at 90 days. Hemorrhagic stroke transformation prevalence in bad outcome group (29%) was possibly inflated by the small sample size. In our sample, only one patient (2.9%) had hypoplasia of cA1, which is slightly lower than that reported in the literature, possibly due to the small sample size [14]. In addition, in the present study, we did not include posterior circulation collaterals, which, in some cases, could compensate for the insufficiency of anterior collaterals. Further studies are needed to assess different CoW variations with varying clot locations to create a robust predictive model for patients undergoing MTE.

Long-term outcome prediction after distal ICA occlusion treatment with MTE is vital for a patient’s further prognosis. Our study chose NIHSS evaluation after seven days, as clinical fluctuations in stroke patients’ neurological state in the first 24 h after MTE are well documented. The evaluation of NIHSS after seven days has a higher specificity and sensitivity than NIHSS after 24 h (83.8% vs. 80.1% and 89.1% vs. 80.4%, respectively) for predicting good outcomes (mRs 0–2 after 90 days) [15].

The narrowest diameter of the cA1 and AComA complex could be used in clinical practice in the case of distal ICA T occlusion to determine which patient group is the most sensitive to the ischemic changes timewise and to predict patient outcomes. Determining patients with narrow diameters could allow for a better selection of patients with a greater risk of larger ischemic areas and tailor the logistics needed to ensure the best possible outcomes for patients with a greater risk of fast-progressing ischemic stroke.

According to our data, a cut-off of 1.1 mm can be used for determining the diameter of the anterior communicating artery or contralateral A1 as being narrow or sufficiently wide.

## 5. Conclusions

In conclusion, patients with distal ICA occlusion and the narrowest diameter of the AComA/cA1 complex smaller than 1.1 mm tended to have poorer clinical outcomes after MTE. As this anatomic feature can be determined in preprocedural imaging studies, it can be used as a prognostic marker to guide patient selection. Further studies with larger sample sizes are needed.

## Figures and Tables

**Table 1:** Summary statistics. In the case of quantitative variables with normal distribution, the mean ± standard deviation is given. Median and IQR [Q1–Q3] are shown for quantitative variables not distributed normally. In the case of categorical variables, the number of observations and percentages are given.

	7-Day NIHSS ≤ 6 (*n* = 28)	7-Day NIHSS > 6 (*n* = 42)	*p*-Value	n Missing
Female, n (%)	20 (71.4)	21 (50.0)	0.0882	0
Age, years (mean ± SD)	72 ± 11	73 ± 10	0.5933	0
Time from last seen well to punction, min (median [Q1–Q3])	160 [185–250]	189 [220–330]	0.2056	3
Narrowest segment of AComA/cA1 complex, mm (mean ± SD)	1.56 ± 0.63;	1.13 ± 0.58;	0.0043	0
ASPECT score on admission, points (median [Q1–Q3])	8 [9–10]	7 [9–10]	0.3150	0
NIHSS score on admission, points (median [Q1–Q3])	17.5 [15.5–21.5]	19 [14–21]	0.8177	0
Intravenous recombinant tissue plasminogen activator, n (%)	15 (53.6)	17 (40.5)	0.3324	0
Primary arterial hypertension, n (%)	7 (25.0)	12 (28.6)	0.7152	36
Heart failure, n (%)	4 (14.3)	8 (19.0)	1.0000	36
Diabetes mellitus, n (%)	2 (7.1)	2 (4.8)	0.5672	37
Hemorrhage on follow-up CT, n (%)	2 (7.1)	12 (28.6)	0.0674	37
Seven-day mortality, n (%)	6 (8.6)			
Hemorrhagic transformation of stroke PH2, n (%)	5 (7.1)			

**Table 2:** *p*-values for comparison of 7-day NIHSS and the narrowest diameter by the collateral grade.

	Collateral Grade			Kruskal–Wallis Test
	0 vs. 1	0 vs. 2	1 vs. 2	
7-day NIHSS	0.966	0.030	0.007	<0.001
Narrowest diameter of AComA/cA1 complex, mm	0.354	<0.001	0.014	<0.001

**Table 3:** The estimates of parameters and odds ratios of univariate logistic regressions.

	Odds Ratio	Odds Ratio 95% CI		*p*-Value
Narrowest segment diameter of AComA/cA1 complex, mm	0.29	0.117	0.723	0.0079
Age	1.013	0.967	1.061	0.5877
Gender (female)	0.400	0.144	1.108	0.0780
Time from last seen well to groin puncture, minutes	1.001	0.998	1.004	0.3699
Intravenous recombinant tissue plasminogen activator, Yes/No	0.589	0.225	1.547	0.2829

## Data Availability

The data presented in this study are available on request from the corresponding author due to hospital policy for patient data.

## Supplementary Materials

The following supporting information can be downloaded at: https://www.mdpi.com/article/10.3390/medicina60111749/s1, Figure S1: Shows the relationship between the Time from the last well seen to groin access and the 7-day NIHSS. No statistically significant relationship was found between the Time from the last well seen to punction and the 7-day NIHSS. Figure S2: Relationship between ASPECT score on admission and 7-day NIHSS. The correlation between ASPECT on score admission and 7-day NIHSS is non-significant (Spearman correlation coefficient −0.21, *p*-value = 0.0875). Figure S3: shows the relationship between baseline NIHSS and 7-day NIHSS. There was no correlation (Spearman correlation coefficient 0.08, *p*-value = 0.4982). Figure S4: shows the relationship between the Narrowest diameter of the AComA/cA1 complex and the Baseline NIHSS. No correlation was found. The Spearman correlation coefficient is 0.0228, and the *p*-value is 0.8512.
