# Supplementary material for: Association of Narrow Anterior Communicating Artery or Contralateral A1 Segment with Poor Outcomes After Mechanical Thrombectomy
Source: Medicina (Kaunas). 2024 Oct 24;60(11):1749. doi: 10.3390/medicina60111749 (PMC11596048; doi:10.3390/medicina60111749)
Supplement: Supplementary file 1 [file medicina-60-01749-s001.zip › Supplementary materials.pdf]

medicina

MDPI

Supplementary materials

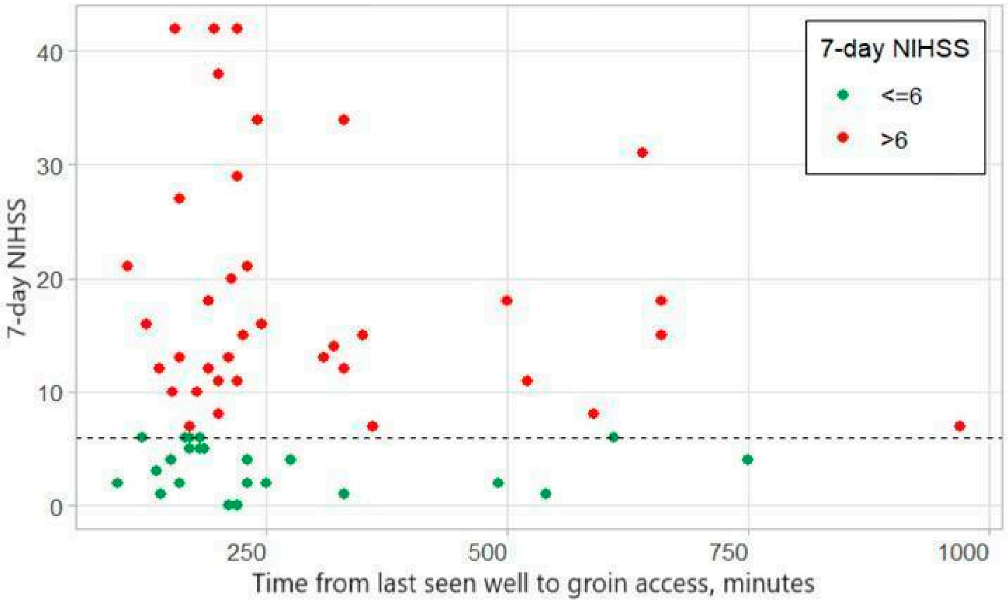

Figure S1. shows the relationship between the Time from the last well seen to groin access and the 7-day NIHSS. No statistically significant relationship was found between the Time from the last well seen to punction and the 7-day NIHSS.

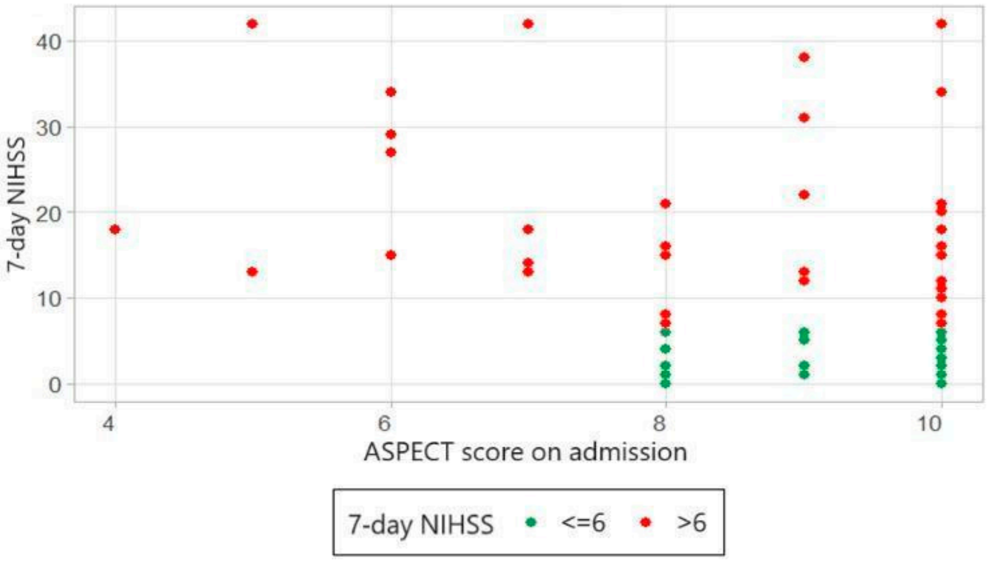

Figure S2. Relationship between ASPECT score on admission and 7-day NIHSS. The correlation between ASPECT on score admission and 7-day NIHSS is non-significant (Spearman correlation coefficient -0.21, p-value = 0.0875).

The correlation between ASPECT on score admission and 7-day NIHSS was non-significant (Spearman correlation coefficient -0.21, p-value = 0.0875) (Figure S2). The relationship between ASPECTS on admission and seven-day NIHSS showed no statistically significant interaction. Still, a trend can be seen - a good ASPECT score does not predict a good outcome (7-day NIHSS <= 6), while a poor ASPECT score (<=7) could predict a poor outcome (Figure 5).

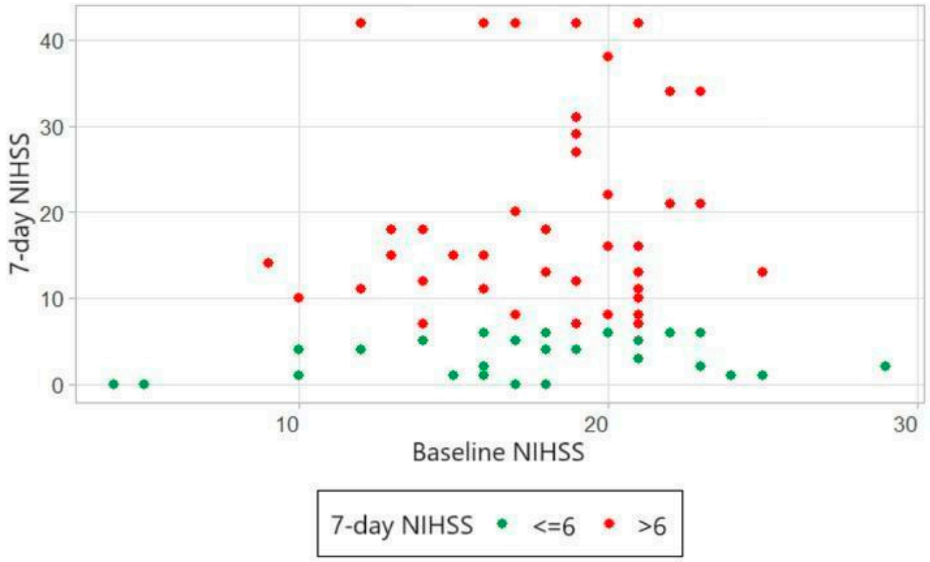

Figure S3 shows the relationship between baseline NIHSS and 7-day NIHSS. There was no correlation (Spearman correlation coefficient 0.08, p-value = 0.4982).

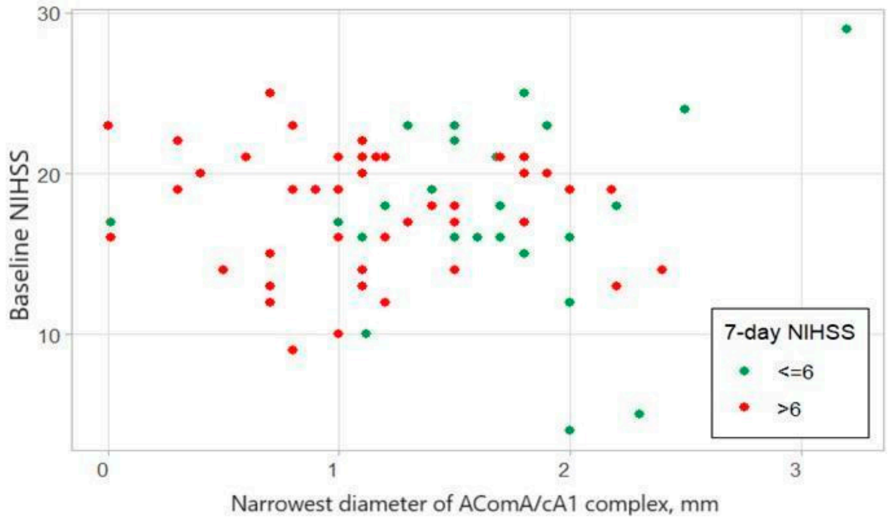

Figure S4. shows the relationship between the Narrowest diameter of the AComA/cA1 complex and the Baseline NIHSS. No correlation was found. The Spearman correlation coefficient is 0.0228, and the p-value is 0.8512.
